# Rate of red blood cell destruction varies in different strains of mice infected with *Plasmodium berghei-ANKA *after chronic exposure

**DOI:** 10.1186/1475-2875-8-91

**Published:** 2009-05-05

**Authors:** Gideon Kofi Helegbe, Nguyen T Huy, Tetsuo Yanagi, Mohammed N Shuaibu, Akiko Yamazaki, Mihoko Kikuchi, Michio Yasunami, Kenji Hirayama

**Affiliations:** 1 Department of Immunogenetics, Institute of Tropical Medicine (NEKKEN), Nagasaki University, 1-12-4 Sakamoto, Nagasaki 852-8523, Japan; 2 Department of Biochemistry and Molecular Medicine, SMHS, UDS, Tamale, Ghana; 3 Animal Research Center for Tropical Infections, Institute of Tropical Medicine (NEKKEN), Nagasaki University, 1-12-4 Sakamoto, Nagasaki 852-8523, Japan; 4 Center for International Collaborative Research, Nagasaki University, 1-12-4 Sakamoto, Nagasaki 852-8523, Japan; 5 Global COE program, Nagasaki University, Japan

## Abstract

**Background:**

Severe malaria anaemia in the semi-immune individuals in the holo-endemic area has been observed to occur at low parasite density with individual variation in the responses. Thus the following has been thought to be involved: auto-immune-mediated mechanisms of uninfected red blood cell destruction, and host genetic factors to explain the differences in individual responses under the same malaria transmission. In this study, the extent of red blood cell (RBC) destruction in different strains of semi-immune mice model at relatively low parasitaemia was studied.

**Methodology:**

To generate semi-immunity, four strains of mice were taken through several cycles of infection and treatment. By means of immunofluorescent assay and ELISA, sera were screened for anti-erythrocyte auto-antibodies, and their relationship with haematological parameters and parasitaemia in the strains of semi-immune mice was investigated.

**Results:**

Upon challenge with *Plasmodium berghei *ANKA after generating semi-immune status, different mean percentage haemoglobin (Hb) drop was observed in the mice strains (Balb/c = 47.1%; NZW = 30.05%; C57BL/6 = 28.44%; CBA = 25.1%), which occurred on different days for each strain (for Balb/c, mean period = 13.6 days; for C57BL/6, NZW, and CBA mean period = 10.6, 10.8, 10.9 days respectively). Binding of antibody to white ghost RBCs was observed in sera of the four strains of semi-immune mice by immunofluorescence. Mean percentage Hb drop per parasitaemia was highest in Balb/c (73.6), followed by C57BL/6 (8.6), CBA (6.9) and NZW (4.0), p = 0.0005. Consequently, auto-antibodies level to ghost RBC were correlated with degree of anaemia and were highest in Balb/c, when compared with the other strains, p < 0.001.

**Conclusion:**

The results presented in this study seem to indicate that anti-RBC auto-antibodies may be involved in the destruction of uninfected RBC in semi-immune mice at relatively low parasite burden. Host genetic factors may also influence the outcome of auto-immune mediated destruction of RBC due to the variation in Hb loss per % parasitaemia and differences in antibody titer for each semi-immune mice strain. However, further studies at the molecular level ought to be carried out to confirm this.

## Background

Malaria continues to claim the life of millions in the tropics and it is reported that 1.5–2.7 million deaths are observed annually mostly due to *Plasmodium falciparum *[1]. Individuals in the endemic regions become semi-immune as a result of the repeated infection [2]. Despite being semi-immune, a significant proportion of these individuals develop the severe forms of malaria disease leading to high mortality and morbidity, with severe malaria anaemia (SMA) as one of the leading causes [3]. However, much remains to be understood of the pathogenesis of SMA.

Central to the proposal to explain the pathogenesis of SMA is the destruction of high numbers of uninfected red blood cells (uRBC) compared with the infected RBC (iRBC) [4], due to the consistent observation of SMA at relatively low parasite burdens of semi-immune individuals in malaria endemic areas [5]. Jakeman *et al *used a mathematical method to evaluate that with one destroyed iRBC, there is 10 destructed uRBCs [6]. The phenomenon of high uRBC destruction at low parasitaemia in the semi-immune is still unclear, but phagocytic cells and/or CD4^+ ^T lymphocytes are thought to play a role [4]. Also, inadequate reticulocyte response has been proposed as being a contributory factor to the SMA, due to an abnormal bone marrow cellularity reflected by low reticulocyte counts in SMA patient [7].

Another process that contributes to the destruction of uRBC is the mechanical mechanism, as indicated by the role of auto-antibodies [8,9]. Even though elevated anti-erythrocyte ghost antibody levels have been demonstrated to be associated with human malaria infections [10], its association with anaemia and host genetic factors has not been clarified in the semi-immune. Anti-erythrocyte auto-antibodies reacting with the surface of normal or acetone fixed human erythrocytes have also been reported to occur in *P. falciparum *patients' sera [11,12] and are thought to be at least in part responsible for the anaemia frequently seen in acutely infected *P. falciparum *patients. Using Direct Coombs antiglobulin test, previous studies proposed a relationship of anti-RBC antibodies in the anaemia seen in *P. falciparum *infections [13,14].

Although the role of auto-immune mechanism in uRBC destruction resulting in anaemia during malaria has been debated for some time, it is still controversial. While some studies have implicated auto-antibodies such as IgM, IgG and IgA classes [8,15-18], as having specificity toward uninfected and infected RBCs, thus playing an auto-immune mediated mechanism of uRBC destruction, and others do not [19]. Thus using the rodent model the association between level of auto-antibodies against uRBC ghost and degree of anaemia at low parasite burden in the semi-immune was investigated. Rodent model of SMA as developed by Evans *et al *[4] are uncomplicated by excessive parasite burdens. In contrast, naïve murine malaria infections are hyperparasitaemic, thereby making the associated haemolytic anaemia not be reflective of SMA in the human populations.

Since severe malaria has been found to vary from one individual to another [20], with the implication of host genetic factors, due to variation in number of infected erythrocytes and spleen size in the naïve murine malaria [21,22], the role of strain specificity in auto immune mediated mechanism of uRBC destruction in the different strains of chronic infected mice was also investigated. Studies have shown that there is a differential level of auto-antibodies in other diseases such as auto-immune haemolytic anaemia in mice strains [23,24].

## Methods

### Mice, malaria infections and profiles of SMA

Four strains of mice BALB/c, C57BL/6 (B6), CBA and New Zealand White (NZW) aged 8 weeks supplied by SLC laboratories, Fukuoka, Japan, were injected intraperitoneally (i.p.) with 10^4 ^*Plasmodium berghei *ANKA-infected RBCs. Parasitaemia and reticulocyte levels were monitored every two days by Giemsa-stained thin blood film and are expressed as a percentage of more than 500 RBCs. Haemoglobin (Hb) was measured in a 96-well plate at 570 nm on Bio-Rad Model 3550 Micro plate Reader as previously described [25]. Four microliter (4 μL) of tail-vein blood was suspended in 1 mL Drabkin reagent (Sigma, St Louis, MO) and absorbance measured, and is expressed as a percentage of baseline levels. Laboratory and animal practices of the Animal Center of Institute of Tropical Medicine (NEKKEN), Nagasaki were adhered to, after the approval from the local ethics committee for animal care and research was obtained.

### Generation of semi-immune mice and harvesting of serum

**Figure 1:**
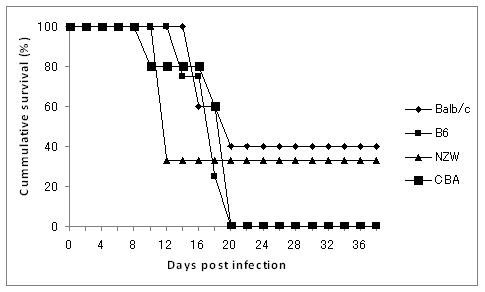
**Cumulative survival curve of semi-immune mice**. Death of semi-immune mice were monitored after challenge with 10^4 ^*P. berghei *ANKA following attainment of semi-immune status in the mice strains without treatment. Balb/c, n = 5; B6, n = 4; NZW, n = 3; CBA, n = 5.

This was a modified method as described elsewhere [4]. Four strains of infected mice were treated at day 6 after infection with chloroquine/(10 mg/kg intraperitoneally) and pyrimethamine (10 mg/kg intraperitoneally) daily for 6 days. During subsequent rounds of infection, mice were rested for two weeks before being rechallenged with 10^4 ^*P. berghei *ANKA, then monitored and drug-cured prior to parasitemias reaching 5%. Mice underwent seven to eight cycles of drug-cured infection before finally being challenged with 10^4 ^*P. berghei *parasites without treatment. Preliminary studies in the laboratory, in which mortality occurred beyond some minimum days after infection (Figure 1) and data from other studies, in which complications arose as a result of parasitaemia between 25 and 75% [26], influenced the time blood harvested for serum. About 100 μL of blood was collected *via *eye vein during the 7^th ^and 8^th ^cycle of infection at low Hb. Two weeks after treatment (7^th ^cycle), blood was collected again for serum. The sera were stored at -30°C until used. The whole set of experiments was performed twice and pooled data are presented.

### Preparation of red blood cell (RBC) white ghost membrane

The method used here was based on a previously described one [27] with some modifications. Briefly, heparinized blood (0.5 mL) from uninfected mice was washed with phosphate buffer saline (PBS), pH 7.4 and later haemolysed in hypotonic phosphate buffer (5 mM, pH 8.0). After vigorous shaking, the haemolysate was washed twice for 20 minutes at 15,000 rpm. The supernatant was removed by aspiration. The membranes were washed six times with the same haemolysate buffer until the pellet became white, and then washed 2–3 times with Tris-HCl (50 mM, pH 7.2) and finally in PBS. Antigen concentration was determined by BCA protein assay kit (Product number 23227, Pierce Biotechnology, Rockford, USA).

### Screening of sera from semi-immune for antibody binding to RBC membrane

Immunofluorescence assay (IFA) was used to check antibody binding to RBC membrane. RBC white ghost membrane prepared above was used as antigen to coat the IFA slides, fixed in cold acetone and washed in PBS. Goat serum (Chemicon International, CA) diluted 1:100, was used for blocking and incubated at room temperature (RT) for 30 minutes. After washing the goat serum with PBS, the serum samples (primary antibody) were added at different dilutions and incubated at RT for 3 hours. Washing was done thrice in PBS and secondary antibody goat anti-mouse IgG-FITC (Sigma-Aldrich, St Louis, Missouri, USA) diluted 1:50, was added to the slides and incubated for an hour in the dark at RT. The slides were later washed thrice in PBS and observed under fluorescence microscope.

### Antibody titer measurement using ELISA

This was a modified method as described previously [28]. The RBC white ghost membrane was used as antigen at a protein concentration of 2 μg in 100 μl of coating buffer (pH 9.6) per well to coat polystyrene plates (Lot number 091611, Nunc, Copenhagen, Denmark) at 4°C overnight. The plates were washed thrice with 0.05% Tween-20-PBS, then optimum blocking conditions for non-specific binding was achieved using 300 μl per well of 0.1% blocking reagent (lot number 13945300, Roche Diagnostics, Mannheim, Germany) -0.1% Tween-20/PBS, pH 7.2, and incubated for 1 hour at 37°C. Plates were washed thrice with PBS containing 0.05% Tween-20. The antigen in coated plates was then reacted with the serum samples obtained from non-infected (as negative control) and infected mice at 1/40 dilutions, in duplicates. After three hours incubation at 37°C, plates were washed five times with 0.05% Tween-20/PBS. Later, 100 μL of horse radish peroxidase (HRP)-conjugated goat anti-mouse IgG (Southern Biotechnology, Birmingham, AL) was added to each well and incubated for 1 hour at 37°C, then washed five times with 0.05% Tween-20/PBS. For colour development, 3, 3', 5, 5'-tetramethylbenzidine (TMB, Catalogue number SK-4400, Vector Laboratories, CA, USA) was used and prepared according to the manufacturer's instructions. The reaction was then interrupted at 30 minutes by the addition of 50 μl 1N H_2_SO_4_. Absorbance was read at 450 nm in EIA-reader (Bio-Rad, Hercules, CA)

### Statistical analysis

Data analysis was done using the GraphPad Prism Version 5.00 for Windows, GraphPad Software, San Diego California, USA, . Data are expressed as the mean with standard error of mean (SEM) unless otherwise stated. Data were log transformed to ensure normal distribution before one-way analysis of variance (ANOVA, with Tukey's post-test), were performed. Pearson correlation analysis was performed on the transformed data of variables to compare the relationship between them. Values were considered significant when p < 0.05.

## Results

### Parasitaemia-time course, profile of severe malaria anaemia and erythropoietic response in the semi-immune mice strains

**Figure 2:**
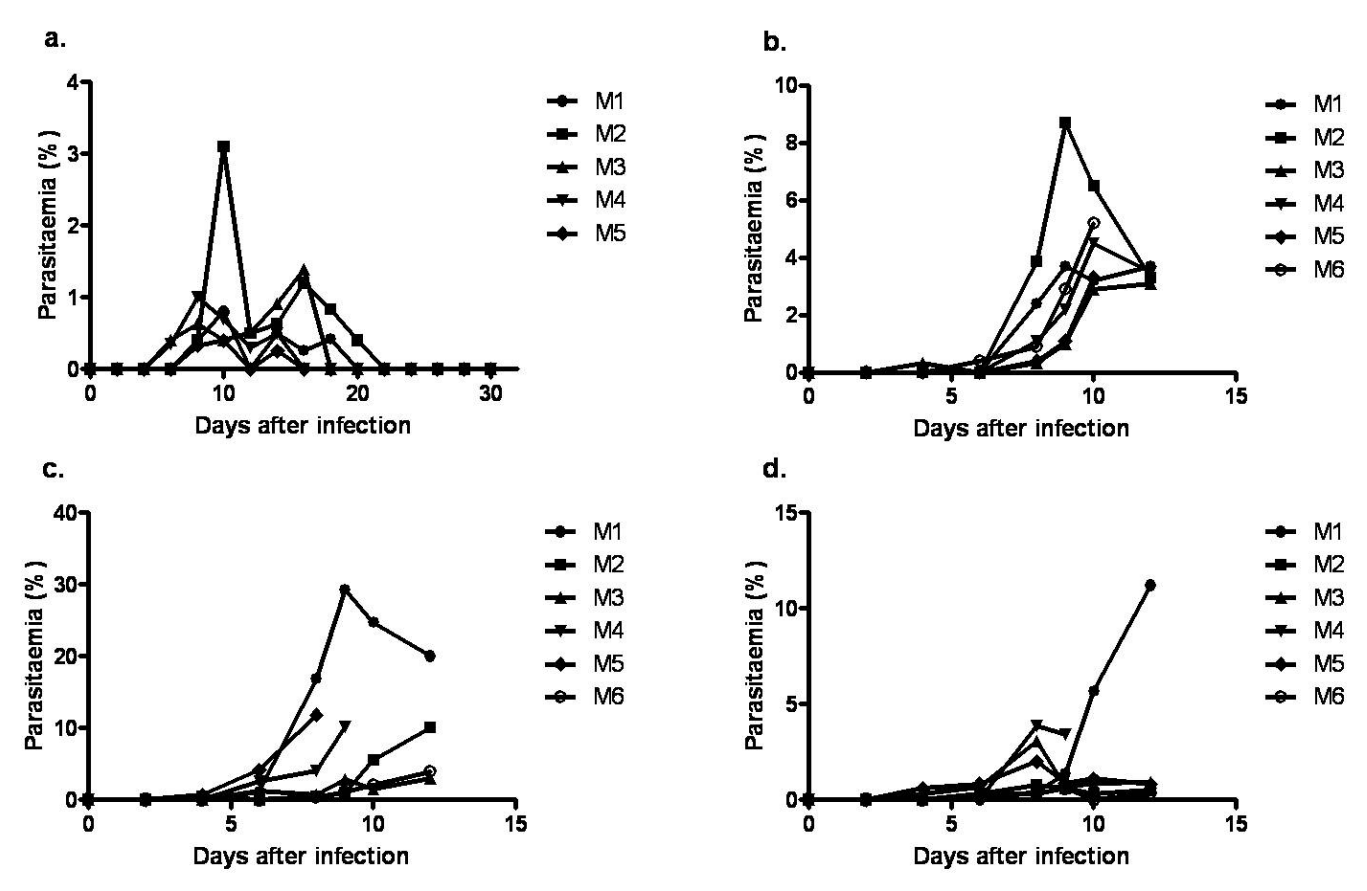
**Parasitaemia time course in the semi-immune mice**. Representative data of infected semi-immune mice (a) Balb/c, n = 5 (b) B6, n = 6 (c) NZW, n = 6 and (d) CBA mice, n = 6 following infection with 10^4 ^*P. berghei *ANKA during the final cycle without treatment.

Four strains of mice (Balb/c, B6, NZW and CBA) were taken through several cycles of infection with 10^4 ^*P. berghei *ANKA followed by pyrimethamine and chloroquine treatment to generate semi-immune status. Upon challenge with 10^4 ^*P. berghei *ANKA after generating semi-immune status (i.e. after the final cycle), similar parasitaemia profiles were observed in different cycles of infection in the same strain, but different responses were observed in the strains (Figure 2). Prepatent period was four days in Balb/c and two days in B6, NZW and CBA. Also there were two peaks of parasitaemia exhibited by the individual Balb/c mice during recovery (Figure 2a). The first peak (0–3.1% parasitaemia) was observed between days 4 and 12 and the second peak (0–1.2% parasitaemia) between days 12 and 22. Individual variation in parasitaemia was also observed in the other semi-immune mice, but at relatively higher parasitaemia, Figures 2b–d. Much higher parasitaemia in the individual mice were observed in the NZW (Figure 2d). Even though some mice appeared to resolve their parasitaemia (Figures 2b–d), they could not fully recover and most of them died.

**Figure 3:**
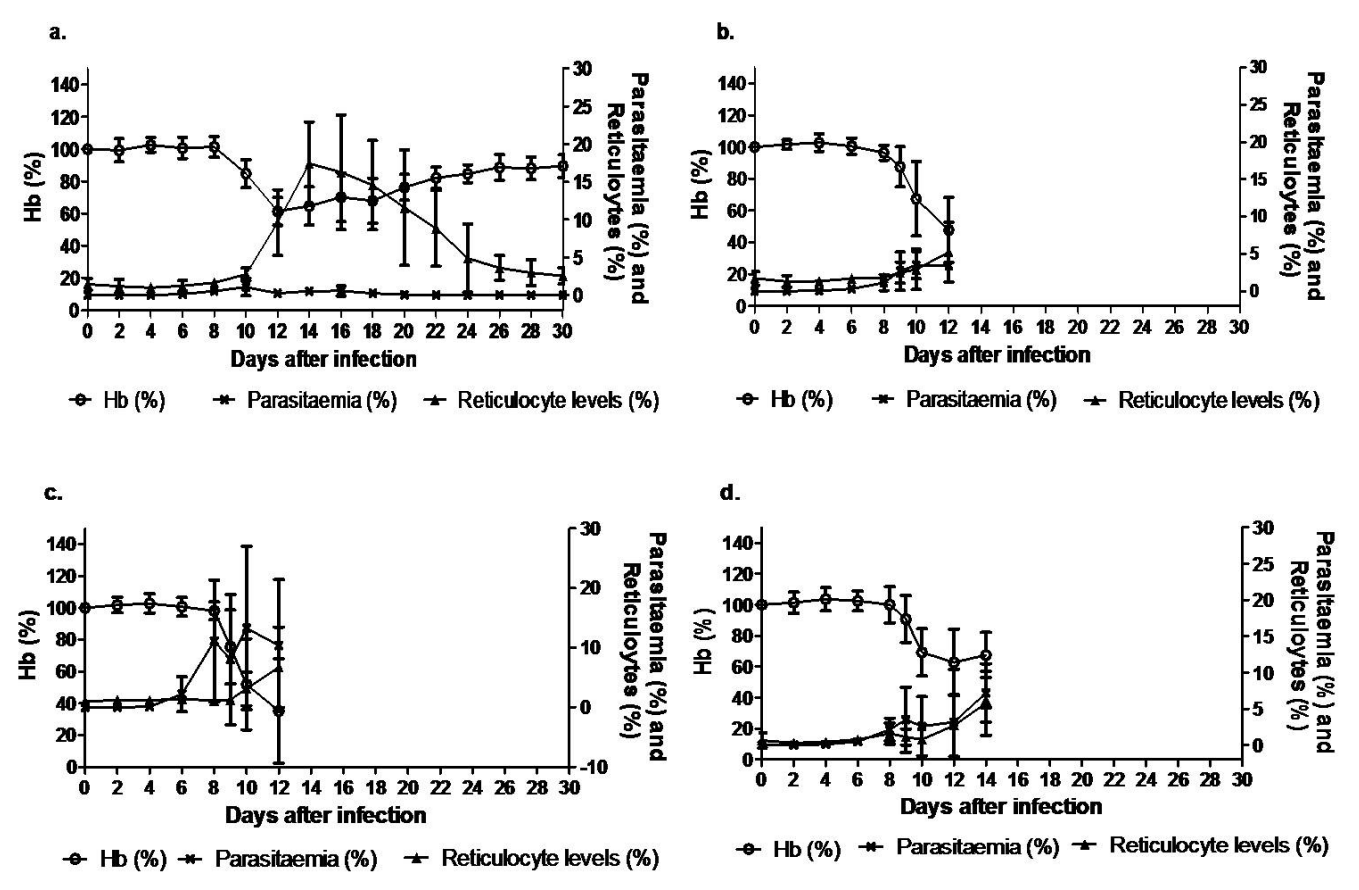
**Profile of malaria anaemia in the semi-immune mice**. Mean parasitaemias, reticulocyte levels and Hb in semi-immune mice strains (a) Balb/c, n = 5 (b) B6, n = 11 (c) NZW, n = 8 and (d) CBA, n = 12, after *P. berghei *ANKA infection during the final cycle. These are pooled data from 2 separate experiments and data are represented as mean ± SD.

**Figure 4:**
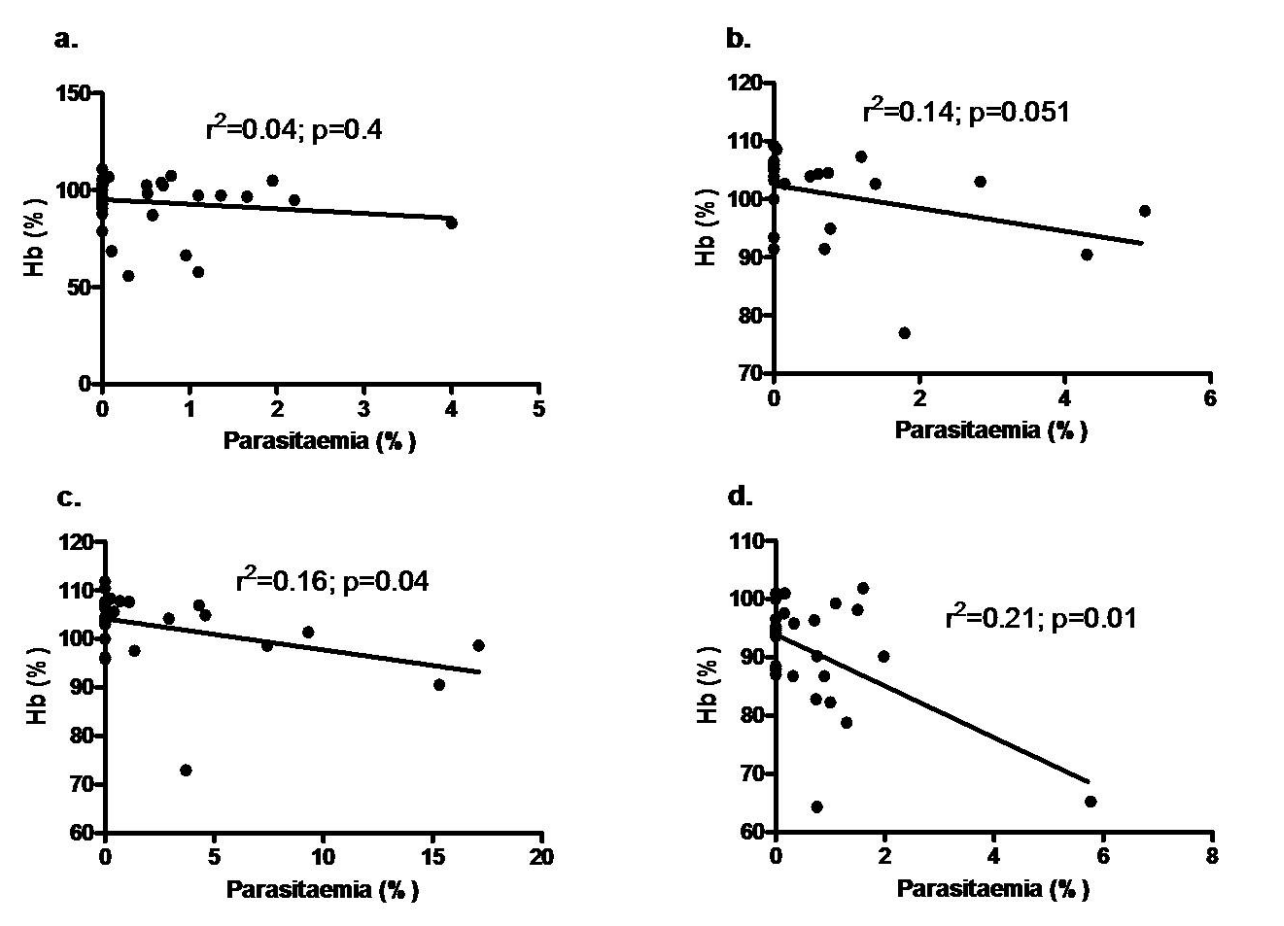
**Relationship between Hb and parasitaemia in semi-immune mice strains**. Mice were infected with 10^4 ^*P. berghei *ANKA to generate semi-immune status in the mice strains. Hb was determined (see text for procedure) and parasitaemia every two days. Hb and parasitaemia were compared in the combined cycles (1–6) to examine the extent of their relationship. (a) Balb/c, n = 5 (b) B6, n = 6, (c) NZW, n = 6 (d) CBA, n = 6. Hb, haemoglobin. These are values of one experiment.

The kinetic profiles of mean blood haemoglobin, reticulocytes, and parasitaemia of four strains were also recorded as shown in Figure 3. One consistent observation made here is that as parasitaemia reached its peak, it was followed by a Hb decrease and an increase of reticulocyte count. Balb/c semi-immune mice resolve their parasitaemia and their Hb improved gradually (Figure 3a). The other strains could not resolve their parasitaemia, hence continued fall in Hb and eventual death (Figure 3b–d). Percentage of mice in each strain that died was similar to that of Figure 1. The correlation between Hb and parasitaemia was poor in Balb/c and B6 (Figure 4a, b) but significant in NZW and CBA (Figure 4c, d), indicating that the degree of anaemia was independent of the level of parasitaemia in Balb/c, B6 while it is correlated in NZW and CBA.

**Figure 5:**
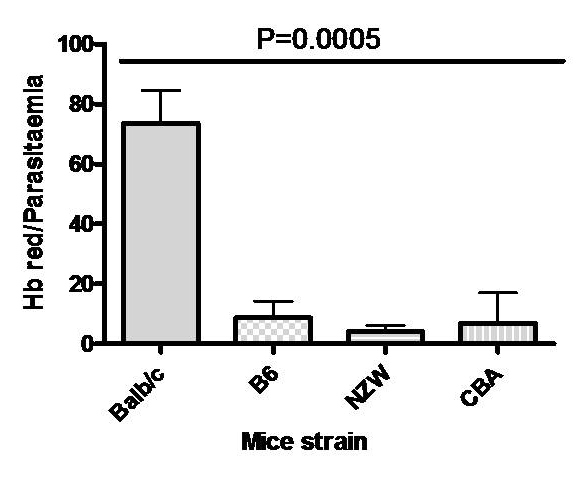
**Percent Haemoglobin drop per parasitaemia in the semi-immune mice strains at later cycles**. Hb reduction (Hb red) for each mouse is the difference in the Hb (baseline, 100%) before first cycle infection and that of Hb_m _during the 7th cycle. To evaluate the extent of Hb reduced per parasitaemia rise, the ratio of the Hb red and parasitaemia at Hb_m _was calculated. The values are pooled data of two separate experiments. Similar observation was made during 8^th ^cycle. Balb/c, n = 11; B6, n = 11, NZW, n = 8, CBA, n = 12. Error bars are standard error of mean (SEM). One way ANOVA was used to analyse data with Tukey post-test, where p values for Balb/c vrs B6, NZW < 0.001, and < 0.01 for CBA. The p values for the others were > 0.05. Hb red, Haemoglobin reduction; Hb_m_, minimum Haemoglobin value.

Haemoglobin levels fell to 47.11%, 28.44%, 30.05% and 25.10% of normal levels in Balb/c, B6, NZW, and CBA, respectively, on days with minimum Hb (Hb_m_) at relatively low parasitaemia, Table 1. Interestingly, Hb reduction in Balb/c, B6 and CBA were at relatively lower mean parasitaemia (<4.0%), compared with NZW mice of mean parasitaemia > 7.0%, p < 0.0001. Moreover, this minimum percentage Hb drop occurred at different period for each semi-immune mice strain, Table 1. During the repeated cycles of infection and treatment, it was observed that relatively higher RBC destruction occurred at later cycles (semi-immune status of mice), but at low parasitaemia in comparison with the first cycle of infection, implying uRBC are destroyed as well and/or inadequate reticulocyte response. With this observation, the extent to which destruction occurred in the semi-immune mice strains at the final cycle and the contribution of parasitaemia was evaluated. It can be seen in Figure 5 that, the mean percentage Hb loss was significantly different statistically in the semi-immune mice strains (p = 0.0005).

**Table 1:** Magnitude of Hb reduction, peak reticulocyte count and peak parasitaemia in the semi-immune mice strain on day minimum Hb was observed

**Parameters**	**Balb/c**	**B6**	**NZW**	**CBA**	**P value^a^**
n	11	11	8	12	-
Mean Hb reduction (95% CI)	47.11^b ^(38.12–56.1)	28.44 (22.82–34.1)	30.05 (16.61–43.5)	25.1 (13.97–36.2)	0.037
Mean Parasitaemia, % (95% CI)	0.64^c ^(0.2–1.1)	3.3 (1.6–4.9)	7.5 (5.0–10.1)	3.1 (0.98–6.25)	< 0.0001
Mean Reticulocyte level, % (95% CI)	12.45^d ^(8.9–15.97)	3.2 (1.9–4.4)	2.0 (0.6–3.4)	3.1 (1.4–4.8)	0.0016
Mean of Reticulocyte level/Hb reduction	0.24^e^	0.11	0.07	0.12	0.003
Mean Period, day (95% CI) at which Hb_m _was observed	13.64^d ^(12.13–15.15)	10.6 (9.95–11.33)	10.86 (9.2–12.5)	10.9 (10.3–11.5)	0.014

Hb reduction is the difference between %Hb on day prior to first cycle infection and treatment, and day on which Hb_m _was observed (blood harvested for serum). The data presented here are pooled results of two separate experiments. ^a^One-way ANOVA with Tukey's post-test. ^b^Significantly higher than CBA; Significantly ^c ^lower, ^d^higher than B6, NZW, CBA; ^e^Significantly higher than B6, NZW. Hb_m_, minimum Hb.

**Figure 6:**
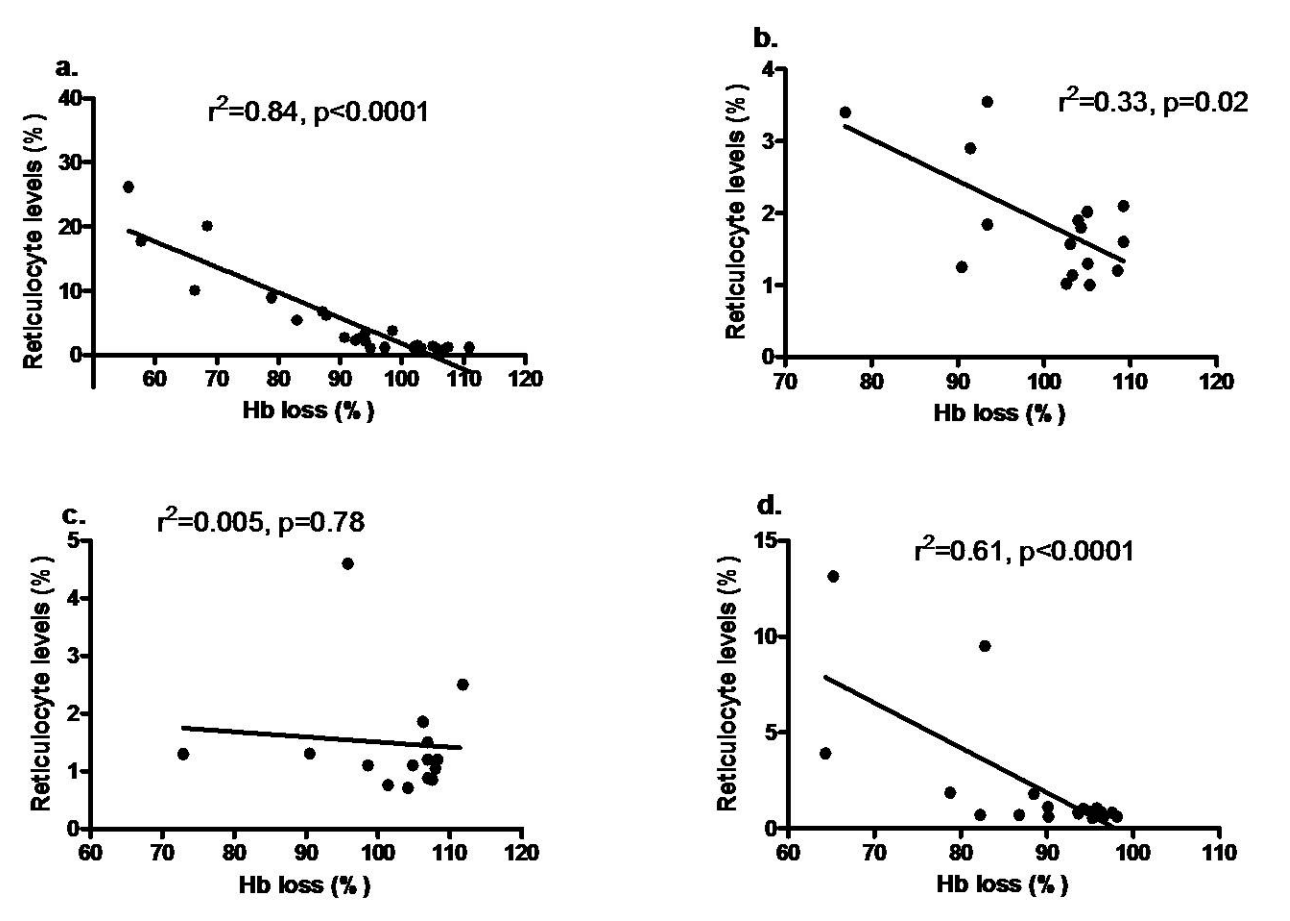
**Relationship between Hb and reticulocyte in semi-immune mice strains**. Mice were infected with 10^4 ^*P. berghei *ANKA to generate semi-immune status in the mice strains. Hb was determined (see text for procedure) and reticulocyte count every two days. Hb and reticulocyte count were compared in the combined cycles (4–6) to examine the extent of their relationship. (a) Balb/c, n = 5 (b) B6, n = 6, (c) NZW, n = 6 (d) CBA, n = 6. Hb, haemoglobin. These are values of one experiment.

Reticulocyte count was estimated in the semi-immune mice to assess extent of erythropoietic response. During the course of infection and treatment to generate semi-immune status, it was observed that reticulocyte production correlated significantly with Hb loss in Balb/c, B6 and CBA, Figure 6. Similar trend was observed in the final cycle where statistically significant correlation of mean Hb reduction levels and reticulocyte levels were observed in the mice strains of Balb/c (r^2 ^= 0.88, p < 0.0001), B6 (r^2 ^= 0.89, p = 0.0014), CBA (r^2 ^= 0.58, p = 0.003), and insignificant in NZW (r^2 ^= 0.30, p = 0.27). The extent of reticulocyte production to Hb loss in Balb/c was two to three times higher than that of B6, CBA and NZW, and statistically significant (Table 1).

### Detection of autoantibody to white ghost RBC membrane

**Figure 7:**
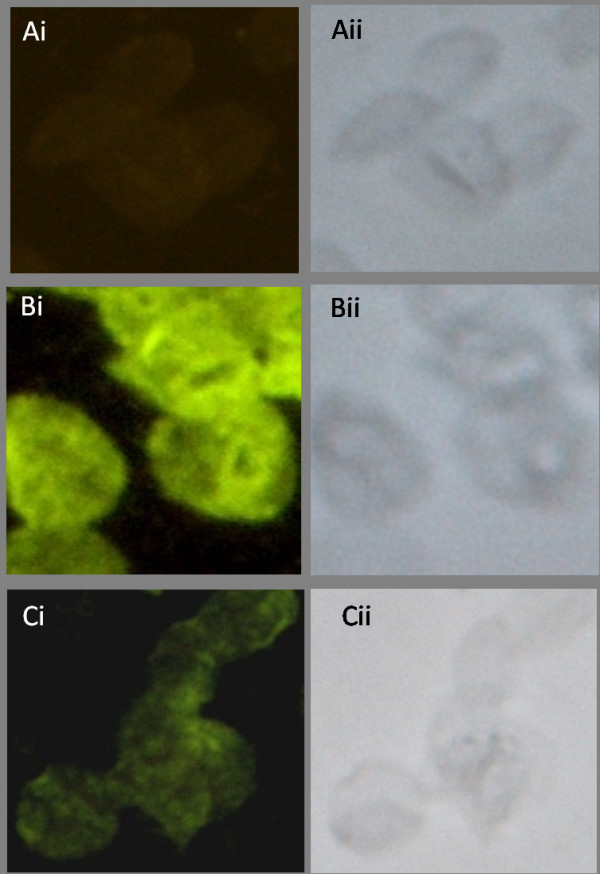
**Reactivity of anti-erythrocyte antibody induced by *Plasmodium berghei *ANKA infection by indirect fluorescent antibody test**. (A) Serum of uninfected mouse reacting with white ghost RBCs from a normal uninfected mouse, under fluorescence i, and bright field, ii. (B) Panel representative of serum from infected semi-immune mice (Balb/c, B6, NZW) and (C) Serum of CBA semi-immune mice reacting with white ghost RBC of a normal uninfected mouse under fluorescence i, and bright field, ii. Serum dilution was at 1:16.

**Figure 8:**
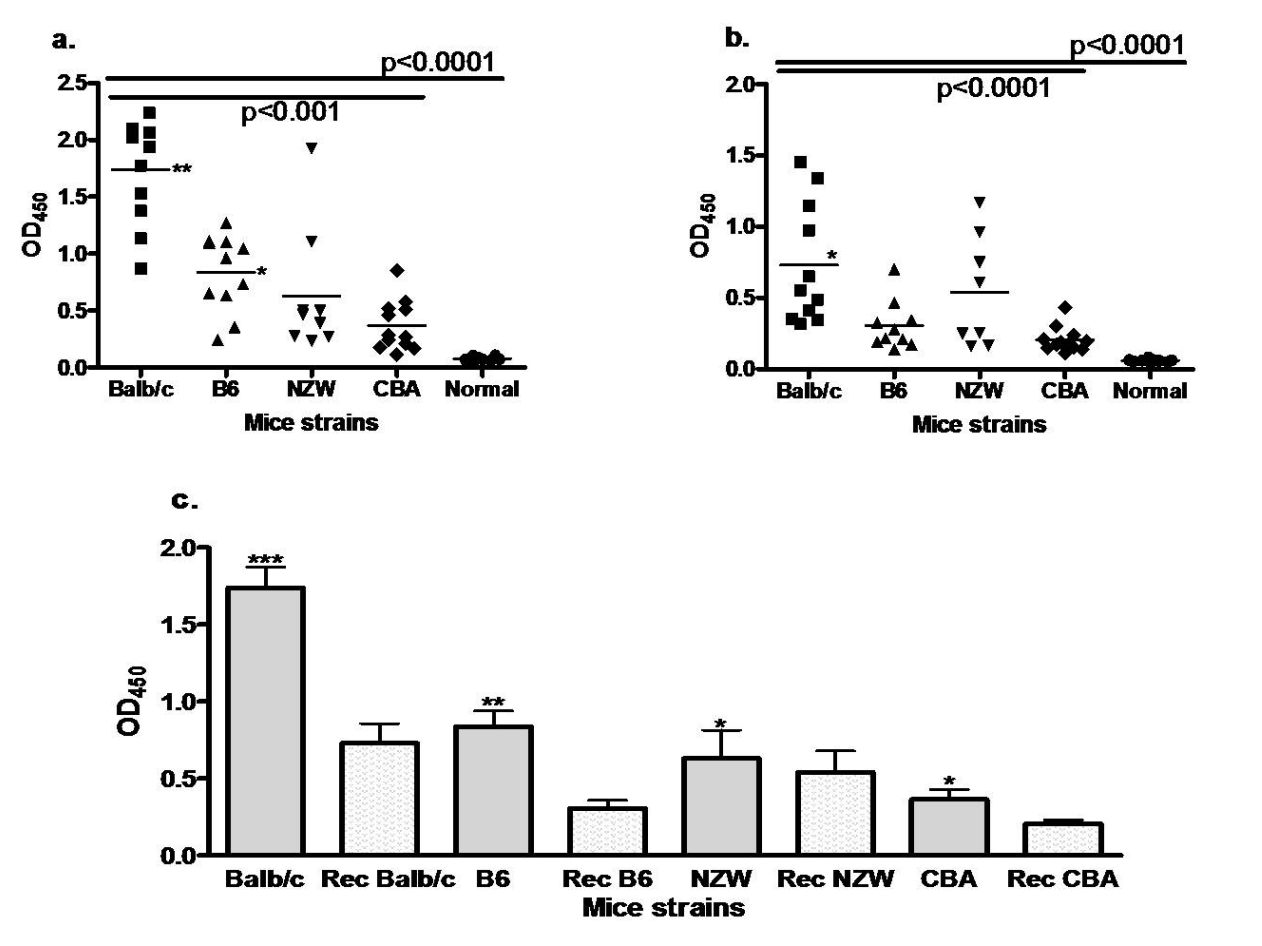
**Antibody level of the sera (1:40) from the semi-immune mice strains against white ghost RBC of an uninfected white ghost RBC**. Sera were harvested in each of semi-immune mice strain at 7^th ^cycle of infection (a) and at recovery (b), and (c) when both are compared. The antibody titer was measured via ELISA. Background values were not higher than 0.06 optical density (OD) units and those for control un-infected sera were slightly higher (mean 0.09). Antibody level in (a) and (b) expressed as individual plots, while the mean is represented by the horizontal bar within the individual plots. (a) **p < 0.001 when compared with the mean antibody titer of the other mice strains; *p < 0.05 when compared with CBA, Tukey's post-test. Balb/c, n = 10; B6, n = 11; NZW, n = 8; CBA, n = 12. (b) *p < 0.001 when compared with B6, CBA. Balb/c, n = 8; B6, n = 9; NZW, n = 7; CBA, n = 11, Normal, n = 9. (c) Error bars are standard error of the mean (SEM). One-way ANOVA was used to analyse data, p < 0.0001. ***/**/*p < 0.001, < 0.05, > 0.05 respectively when that at low Hb with infection is compared with that at recovery for each strain. Balb/c, n = 8; B6, n = 9; NZW, n = 7; CBA, n = 11. Results presented here are pooled data of two separate experiments, with duplicates.

The binding of antibody to white ghost RBC in sera of all the semi-immune mice was observed using IFA as shown in Figure 7. To further understand if antibody level is related to extent of Hb loss, anti-RBC auto-antibodies against white ghost RBC was estimated and observed that antibody level in the sera of the mice strains were significantly higher in Balb/c when compared with the other strains, p < 0.001 (Figure 8a); thus consistent with the observations made in Table 1 regarding amount of Hb loss. To further evaluate the effect of autoantibody at recovery where parasitaemia was not detected, amount of auto-antibody to white ghost RBC in the sera at recovery of the semi-immune mice was estimated and observed that the titer of autoantibody to RBCs within the strains were statistically different, p < 0.0001 (Figure 8b). Furthermore, anti-RBC antibody at recovery was similar to that at low Hb with infection, p > 0.05 for NZW and CBA, whereas that of Balb/c and B6 were significantly different, p < 0.001 and < 0.05 respectively (Figure 8c). Also auto-antibody at recovery for Balb/c was only statistically significant when compared with that of CBA at recovery.

**Figure 9:**
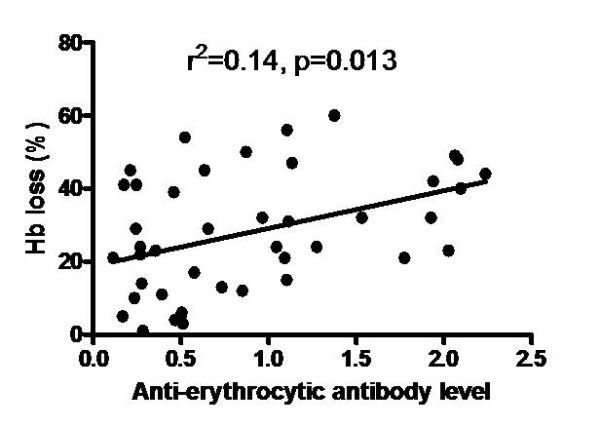
**Correlation of anti-erythrocytic antibody with Hb loss in the semi-immune mice strains**. Extent of Hb loss was estimated at the final cycle after challenging with 10^4 ^*P. berghei *ANKA. Sera were also harvested when minimum Hb was observed at this last cycle, and anti-erythrocytic antibody measured by ELISA. The above are individual values of mice of all the mice strains, n = 42 (Balb/c, n = 11, B6, n = 11; NZW, n = 8; CBA, n = 12), at the final cycle. These are pooled data of two experiments.

To further explore the relationship of auto-antibodies with degree of anaemia, anti-erythrocytic antibody was analysed with extent of Hb loss during the final cycle. This was to understand further the relationship between the two in all the mice strains. It was observed that the correlation was significant (Figure 9), thus indicating that anti-erythrocytic antibody does play a role in the extent of uRBC destruction.

## Discussion

In the early stage of malarial infection, destruction of iRBCs is the primary cause of the anaemia [29]. The severity of anaemia with acute *P. falciparum *malaria correlates with density of parasitaemia [30]. However, in the semi-immune studies have observed that malaria anaemia occurs at low parasitaemia [4,5], and variation in extent of Hb reduction has also been noted in these anaemic individuals. However, the association of this RBC destruction in the semi-immune mice with an immunologic mechanism *via *auto-antibody, and host genetic factors has not been explored. Results from this study shows that auto-antibody may play a role in the destruction of uRBC leading to low Hb in the semi-immune mice at low parasite burden and associated with host genetic factors.

The study here on SMA at low parasitaemia provided a fine opportunity to evaluate extent of uRBC destruction in the semi-immune. The kinetics of blood haemoglobin, reticulocyte levels and parasitaemia showed that Hb improved gradually in Balb/c, even though reticulocyte production in Balb/c was 2–3 times more than the other mice. In as much as inadequate reticulocyte response [7] and destruction of iRBC cannot be excluded, destruction and elimination of uRBC in chronic infected mice may be a major contributory factor resulting in anaemia as observed in another [4] and this study. This is demonstrated during the evaluation of Hb reduction per parasitaemia at the final cycle in the semi-immune mice strains and the observation of Hb loss at a much lower parasitaemia during one of the cycles of infection when compared with the first cycle infection. A recent study has shown that actual parasite numbers may be a major factor in evaluating anaemia than percent parasitaemia [25]. However, in this study only percent parasitaemia was considered, thus further study to estimate the role of actual parasite number in such a study will be interesting. The kinetics and magnitude of reticulocyte production have been observed to be similar in both phenylhydrazine-induced anaemia and *P. berghei *ANKA infected mice [4], suggesting reticulocyte response was adequate. Inadequate reticulocyte response may be a major factor to low Hb in naïve hyperparasitaemic [31] or acute infections. Another possible mechanism to explain for the observed low Hb during Plasmodium infections is the preference of *P. berghei *ANKA for young erythrocytes/reticulocytes [32,33]. Thus, at all levels of parasitaemia, more especially when Hb is low, higher proportions of parasitized reticulocytes than parasitized erythrocytes have been shown to occur [31]. Due to this phenomenon, not enough reticulocytes are able to develop into mature RBC, as both infected and uninfected reticulocytes are cleared [31], hence the persistent low Hb in the chronic infected mice despite compensatory erythropoiesis response to haemolytic anaemia.

The destruction of uRBC may be auto-immune mediated [16] due to the high statistical significant anti-RBC ghost antibodies reported in this study and its significant correlation with anaemia. However, it is suspected that the high auto-antibody mediation could be as a result of the RBC destruction. In that sense a lot of antigens are exposed thus enhancing the synthesis of the antibodies especially in Balb/c. The low parasitaemia observed in the Balb/c seems to indicate that its immunity is much more enhanced compared to the other strains. As a result Balb/c is able to control the parasitaemia growth. The high immune status coupled with the high antibody level in Balb/c appeared to be protective but at a cost, resulting in pathology situation of low Hb. This anti-RBC ghost antibody may lead to sensitization of RBC resulting in immune complex formation during malaria infection at the acute anaemia phase of malaria infection, which has been widely proposed as the cause of RBC destruction [16] and resultant anaemia [34]. Several additional autoantigens have been implicated in the auto-immune disorders occurring during malaria, including modified antigen-antibody complexes [18]. Also, these surface-adherent antigen-antibody complexes initiate complement activation [35,36] inducing a prehaemolytic or a haemolytic condition, as observed in this study. The entire immune complex may be auto-immune responses leading to elimination of RBCs. The observation of continues fall in Hb after parasite clearance following treatment with antimalarial in this study and others [7], in addition to IFA and ELISA results further support the fact that auto-immune mechanisms may be involved to some extent in the low Hb observed at relatively low parasitaemia. Similar observation was made to give explanation for the low Hb during babesiosis infections in cows [28]. In addition to the IFA result in that study [28], higher anti-erythrocytic auto-antibody to ghost RBC was reported in the naturally infected cows in comparison with the non-infected. A contrasting result was, however, obtained in another study, where lack of association between auto-immune mechanism and RBC in chronic malaria was reported [19]. It is not clear if the different parasite strain used could result in this difference, thus this needs to be investigated further.

Previous work showed that depletion of macrophage delayed the clearance of uRBCs in mice, suggesting a role of macrophage in the destruction of uRBCs [4]. At the onset of malaria infection, macrophage activity is crucial to control level of parasitaemia, via eythrophagocytosis, which is enhanced by opsonization with antibodies and other immune reactions like complement [35,36]. However, the over activity can result in pathology (such as low Hb) and sometimes death [37]. Although significantly high anti-RBC autoantibody was observed in the mice strains, which will enhance macrophage activity, it was surprising that comparative Hb drop, was not observed in them as in semi-immune Balb/c. It is possible the macrophage activity may have been impaired or switched off in semi-immune B6, NZW and CBA. The evidence of low Hb drop at relatively higher parasitaemia in these semi-immune strains on one hand and Balb/c on the other could implicate haemozoin; a waste product of haemoglobin may be a contributing factor. In addition to stimulating TNF secretion, it is known to impair macrophage function [38]. The relative higher percent parasitaemia observed in the other semi-immune mice strains other than Balb/c might produce a higher amount of haemozoin, which may impair macrophage function. In addition, haemozoin is also reported to suppress erythropoiesis [39], agreeing well with the data in Figure 3 and Table 1, further supporting that macrophage is suppressed by haemozoin in these strains.

Variation in Hb drop and anti-erythrocytic auto-antibody at low parasitaemia in the semi-immune mice give cause to assume more of host genetic factors are at play. It was realized that in some of the strains more of Hb were lost at relatively much lower parasitaemia, and the possibility of their unique genetic background might play a great role in this various responses. How this affect the variation in Hb loss could be point for further research. This observation goes to establish the fact that despite being exposed to similar plasmodium infections at various times to become semi-immune, the individuals respond differently with some able to withstand the parasite pressure by controlling the parasite growth and others not, leading to high parasitaemia with anaemia and eventually died. It is postulated that the unique genetic background may be responsible in determining how individuals under the same level of malaria transmission in endemic areas respond differently to uRBC destruction at low parasitaemia. It is of interest to note that, the results shown here, reveals that the immune status of the semi-immune appears to delay peak parasitaemia when compared with the naïve status [4,40], by 2–5 days depending on the mice strain, suggesting the immune system of the semi-immune has been developed to some extent in that regard, during the repeated infections and treatment. Also, more especially in the other strains, absence of parasites at recovery could imply that the considerable effect it (parasites) exert on its host RBC, which eventually lead to similar alterations as seen in oxidatively damaged normal RBC [41,42] are no more. Consequently uRBC destruction is minimized.

The rodent model reported in this study is unique as it enables the study and comparison of RBC destruction in different mice strain at the same time. Similar Hb reduction in the semi-immune Balb/c compares with another study [4], and to the knowledge of the authors those of semi-immune B6, NZW and CBA are the first to be reported here. While, some deaths were observed in this study, none was reported in that by Evans *et al*, [4]. It is not clear if the source of parasite could contribute to this. Also one advantage of the rodent model is that Hb loss at relatively low parasitaemia could be studied, which is similar to humans. However, a disadvantage in the model reported in this study is that Hb loss was just about 50% of baseline, where as Hb values < 50% has been observed in infants [43]. It is possible the mice in this study might have become adults after several cycles of infection and treatment to generate the semi-immune status.

Finally, a study into the possible candidate gene that might be responsible in eliciting the various responses especially of Balb/c on one hand and others such as CBA, by studying into their F1 cross, will be very informative. This will help in understanding further the role of host genetic factors in auto-immune mediated RBC destruction in malaria anaemia at the molecular level.

## Conclusion

Together, results from this study show auto-antibody may play a role in the destruction of uRBC in the semi-immune individuals, as shown in the present mice model. In addition, host genetic factors to some extent influence the outcome of auto-immune mediated mechanism in RBC destruction. This suggests that the host has evolved a mechanism in controlling the degree of RBC destruction, to the benefit of some and detrimental to others. The significance of this study to human malaria of diverse genetic background cannot be overemphasized and warrant further study at the molecular level.

## Competing interests

The authors declare that they have no competing interests.

## Authors' contributions

NTH, AY, MY, KH designed the work with GKH. TY and MNS carried out animal experiment and IFA with GKH. MK designed and carried out the ELISA with GKH. GKH drafted the manuscript with MNS, NTH and KH, who were also involved with data analysis as well extensive revision of the manuscript for intellectual content. NTH and KH supervised the work.

## References

[B1] Breman JG, Alilio MS, Mills A (2004). Conquering the intolerable burden of malaria: what's new, what's needed: a summary. Am J Trop Med Hyg.

[B2] Wipasa J, Elliott S, Xu H, Good MF (2002). Immunity to asexual blood stage malaria and vaccine approaches. Immunol Cell Biol.

[B3] Murphy SC, Breman JG (2001). Gaps in the childhood malaria burden in Africa: cerebral malaria, neurological sequelae, anemia, respiratory distress, hypoglycemia, and complications of pregnancy. Am J Trop Med Hyg.

[B4] Evans KJ, Hansen DS, van Rooijen N, Buckingham LA, Schofield L (2006). Severe malarial anemia of low parasite burden in rodent models results from accelerated clearance of uninfected erythrocytes. Blood.

[B5] Price RN, Simpson JA, Nosten F, Luxemburger C, Hkirjaroen L, ter Kuile F, Chongsuphajaisiddhi T, White NJ (2001). Factors contributing to anemia after uncomplicated falciparum malaria. Am J Trop Med Hyg.

[B6] Jakeman GN, Saul A, Hogarth WL, Collins WE (1999). Anaemia of acute malaria infections in non-immune patients primarily results from destruction of uninfected erythrocytes. Parasitology.

[B7] Phillips RE, Looareesuwan S, Warrell DA, Lee SH, Karbwang J, Warrell MJ, White NJ, Swasdichai C, Weatherall DJ (1986). The importance of anaemia in cerebral and uncomplicated falciparum malaria: role of complications, dyserythropoiesis and iron sequestration. Q J Med.

[B8] Voller A (1974). Immunopathology of malaria. Bull World Health Organ.

[B9] Waitumbi JN, Opollo MO, Muga RO, Misore AO, Stoute JA (2000). Red cell surface changes and erythrophagocytosis in children with severe *Plasmodium falciparum *anemia. Blood.

[B10] Wahlgren M, Berzins K, Perlmann P, Bjorkman A (1983). Characterization of the humoral immune response in *Plasmodium falciparum *malaria. I. Estimation of antibodies to P. falciparum or human erythrocytes by means of microELISA. Clin Exp Immunol.

[B11] Rosenberg EB, Strickland GT, Yang SL, Whalen GE (1973). IgM antibodies to red cells and autoimmune anemia in patients with malaria. Am J Trop Med Hyg.

[B12] Zouali M, Druilhe P, Gentilini M, Eyquem A (1982). High titres of anti-T antibodies and other haemagglutinins in human malaria. Clin Exp Immunol.

[B13] Facer CA, Bray RS, Brown J (1979). Direct Coombs antiglobulin reactions in Gambian children with *Plasmodium falciparum *malaria. I. Incidence and class specificity. Clin Exp Immunol.

[B14] Woodruff AW, Ansdell VE, Pettitt LE (1979). Cause of anaemia in malaria. Lancet.

[B15] Adner MM, Altstatt LB, Conrad ME (1968). Coombs'-positive hemolytic disease in malaria. Ann Intern Med.

[B16] Zuckerman A (1964). Autoimmunization and Other Types of Indirect Damage to Host Cells as Factors in Certain Protozoan Diseases. Exp Parasitol.

[B17] Kreier J, Shapiro H, Dilley D, Szilvassy IP, Ristic M (1966). Autoimmune reactions in rats with *Plasmodium berghei *infection. Exp Parasitol.

[B18] Topley E, Knight R, Woodruff AW (1973). The direct antiglobulin test and immunoconglutinin titres in patients with malaria. Trans R Soc Trop Med Hyg.

[B19] Wu YL, Yu Q, Li WL, Liu EX (1989). Studies on the mechanism of anemia in rodent malaria. Proc Chin Acad Med Sci Peking Union Med Coll.

[B20] Hananantachai H, Patarapotikul J, Ohashi J, Naka I, Looareesuwan S, Tokunaga K (2005). Polymorphisms of the HLA-B and HLA-DRB1 genes in Thai malaria patients. Jpn J Infect Dis.

[B21] Eling W, van Zon A, Jerusalem C (1977). The course of a *Plasmodium berghei *infection in six different mouse strains. Z Parasitenkd.

[B22] Adun EH, Williams JS, Meroney FC, Hutt G (1965). Pathophysiology of *Plasmodium berghei *infection in mice. Exp Parasitol.

[B23] Caulfield MJ, Stanko D, Calkins C (1989). Characterization of the spontaneous autoimmune (anti-erythrocyte) response in NZB mice using a pathogenic monoclonal autoantibody and its anti-idiotype. Immunology.

[B24] Menshikov I, Beduleva L (2008). Evidence in favor of a role of idiotypic network in autoimmune hemolytic anemia induction: theoretical and experimental studies. Int Immunol.

[B25] Lamb TJ, Langhorne J (2008). The severity of malarial anaemia in *Plasmodium chabaudi *infections of BALB/c mice is determined independently of the number of circulating parasites. Malar J.

[B26] Langhorne J, Quin SJ, Sanni LA (2002). Mouse models of blood-stage malaria infections: immune responses and cytokines involved in protection and pathology. Chem Immunol.

[B27] Huy NT, Serada S, Trang DT, Takano R, Kondo Y, Kanaori K, Tajima K, Hara S, Kamei K (2003). Neutralization of toxic heme by *Plasmodium falciparum *histidine-rich protein 2. J Biochem.

[B28] Goes TS, Goes VS, Ribeiro MF, Gontijo CM (2007). Bovine babesiosis: anti-erythrocyte antibodies purification from the sera of naturally infected cattle. Vet Immunol Immunopathol.

[B29] Menendez C, Fleming AF, Alonso PL (2000). Malaria-related anaemia. Parasitol Today.

[B30] Biemba G, Dolmans D, Thuma PE, Weiss G, Gordeuk VR (2000). Severe anaemia in Zambian children with *Plasmodium falciparum *malaria. Trop Med Int Health.

[B31] Cromer D, Evans KJ, Schofield L, Davenport MP (2006). Preferential invasion of reticulocytes during late-stage *Plasmodium berghei *infection accounts for reduced circulating reticulocyte levels. Int J Parasitol.

[B32] Singer I (1954). The course of infection with *Plasmodium berghei *in inbred CF 1 mice. J Infect Dis.

[B33] Collins WE, Jeffery GM, Roberts JM (2003). A retrospective examination of anemia during infection of humans with *Plasmodium vivax*. Am J Trop Med Hyg.

[B34] Owuor BO, Odhiambo CO, Otieno WO, Adhiambo C, Makawiti DW, Stoute JA (2008). Reduced immune complex binding capacity and increased complement susceptibility of red cells from children with severe malaria-associated anemia. Mol Med.

[B35] Helegbe GK, Goka BQ, Kurtzhals JA, Addae MM, Ollaga E, Tetteh JK, Dodoo D, Ofori MF, Obeng-Adjei G, Hirayama K, Awandare GA, Akanmori BD (2007). Complement activation in Ghanaian children with severe *Plasmodium falciparum *malaria. Malar J.

[B36] Goka BQ, Kwarko H, Kurtzhals JA, Gyan B, Ofori-Adjei E, Ohene SA, Hviid L, Akanmori BD, Neequaye J (2001). Complement binding to erythrocytes is associated with macrophage activation and reduced haemoglobin in *Plasmodium falciparum *malaria. Trans R Soc Trop Med Hyg.

[B37] Butcher GA (1996). Malaria and macrophage function in Africans: a possible link with autoimmune disease?. Med Hypotheses.

[B38] Turrini F, Schwarzer E, Arese P (1993). The involvement of hemozoin toxicity in depression of cellular immunity. Parasitol Today.

[B39] Casals-Pascual C, Kai O, Cheung JO, Williams S, Lowe B, Nyanoti M, Williams TN, Maitland K, Molyneux M, Newton CR, Peshu N, Watt SM, Roberts DJ (2006). Suppression of erythropoiesis in malarial anemia is associated with hemozoin in vitro and in vivo. Blood.

[B40] Lou J, Lucas R, Grau GE (2001). Pathogenesis of cerebral malaria: recent experimental data and possible applications for humans. Clin Microbiol Rev.

[B41] Golenser J, Chevion M (1989). Oxidant stress and malaria: host-parasite interrelationships in normal and abnormal erythrocytes. Semin Hematol.

[B42] Hunt NH, Stocker R (1990). Oxidative stress and the redox status of malaria-infected erythrocytes. Blood Cells.

[B43] Ong'echa JM, Keller CC, Were T, Ouma C, Otieno RO, Landis-Lewis Z, Ochiel D, Slingluff JL, Mogere S, Ogonji GA, Orago AS, Vulue JM, Kaplan SS, Day RD, Perkins DJ (2006). Parasitemia, anemia, and malarial anemia in infants and young children in a rural holoendemic *Plasmodium falciparum *transmission area. Am J Trop Med Hyg.

